# Reduced mentalizing in patients with bulimia nervosa and features of borderline personality disorder: A case-control study

**DOI:** 10.1186/s12888-019-2112-9

**Published:** 2019-05-06

**Authors:** Sofia Sacchetti, Paul Robinson, Alexandra Bogaardt, Ajay Clare, Catherine Ouellet-Courtois, Patrick Luyten, Anthony Bateman, Peter Fonagy

**Affiliations:** 1 Research Department of Clinical, Education and Health Psychology, University College London, London, UK; 2 Nutrition Science Group, UCL Division of Medicine, University College London, London, UK; 3 Barnet Enfield and Haringey Mental Health Trust, London, UK

**Keywords:** Bulimia nervosa, Borderline personality disorder, Eating disorder, Mentalizing, Theory of mind, Differentiation-relatedness, Reading the mind in the eyes, Reflective function

## Abstract

**Background:**

Mentalizing, the mental capacity to understand oneself and others in terms of mental states, has been found to be reduced in some mental disorders such as Borderline Personality Disorder (BPD). Some studies have suggested that Eating Disorders (EDs) may also be associated with impairments in mentalizing, but studies have not always yielded consistent results. This is the first study to systematically investigate mentalizing impairments in patients with Bulimia Nervosa (BN) compared with controls. In addition, we investigated whether impairments in mentalizing were related to BPD features, rather than BN per se, given the high comorbidity between BPD and BN.

**Methods:**

Patients with BN (*n* = 53) and healthy controls (HCs; *n* = 87) completed a battery of measures assessing mentalizing including the Reflective Function Questionnaires (RFQ), the Object Relations Inventory (ORI; Differentiation-Relatedness Scales) and the Reading The Mind in The Eyes Test (RMET).

**Results:**

Patients with BN scored significantly lower than HCs on all tests of mentalizing, with moderate to large between-group effect sizes. These differences were partially accounted for by BPD features as assessed with the Zanarini Rating Scale for Borderline Personality Disorder (ZAN-BPD), and partially by bulimic symptoms measured with the Eating Disorder Examination Questionnaire (EDE-Q).

**Conclusions:**

Patients with BN have significantly lower levels of mentalizing as assessed with a broad range of tests compared to HCs. These differences were related to both bulimic symptoms and BPD features. Although further research in larger samples is needed, if replicated, these findings suggest that poor mentalizing may be a significant factor in BN patients and should be addressed in treatment, regardless of the presence of BPD features.

## Background

Bulimia Nervosa (BN) is a debilitating and complex Eating Disorder (ED) characterized by the presence of binging and purging episodes which are generally accompanied by excessive concern about body weight and shape as well as body image disturbance [1, 28].

The onset of BN usually occurs during adolescence or early adulthood. Nonetheless, the disorder tends to persist for several years after onset, with a chronic or intermittent course [1, 8]. In general, EDs are a family of psychopathologies often resistant to treatment with high relapse rates [28, 37]. Relapse remains a significant concern in BN with rates that range between 27.6 and 41% within 2 years after remission [47, 49]. Moreover, the vast majority of relapses occur in the first months after treatment completion [49]. In light of this data, it is important to clarify the core psychological mechanisms that sustain the disorder and that should be addressed during treatment [28].

In this respect, it has been postulated that EDs might develop as a result of emotional difficulties [8]. Indeed, the importance of addressing mood intolerance and emotion dysregulation has been stressed in different treatment protocols including enhanced cognitive behavioural therapy (CBT-E; [27]) and in dialectical behavioural therapy (DBT; [64]).

Moreover, several studies have linked ED symptoms with deficits in mentalizing suggesting that emotional difficulties in this population may arise from a lack of imaginative mental activity about intentional mental states [23, 40, 48, 50]. The term mentalizing indeed refers to the capacity to be aware of both self-experience and interpersonal experience of mental states [4, 7]. In other words, mentalizing implies perceiving and interpreting human behaviours as the result of subjective states and mental processes, such as needs, desires, feelings, thoughts and beliefs. Mentalizing provides the individual with the capacity to understand the representational nature of mind and to distinguish between internal and external reality [34]. This capacity, in turn, is deemed to be essential for recognizing and regulating emotions effectively [5].

BN patients have been shown to demonstrate problems with regard to mentalizing about the self, as expressed in high levels of alexithymia (the inability to label emotions), accompanied by an impaired capacity for symbolization (the capacity to create symbolic representations; [22, 36, 57, 58]). Consistent with these findings, bulimic symptoms have been conceptualized as maladaptive strategies for managing affects in the presence of impaired emotion recognition and regulation [20, 50].

Furthermore, not only do individuals with EDs have difficulty recognizing their own emotional states, but they also show impairments in acknowledging other people’s emotions [17, 39]. In this respect, a recent meta-analysis reported emotion recognition in others to be impaired both in Anorexia Nervosa (AN) and BN patients. However, more severe deficits were found in acute AN, while BN was associated with only a small impairment [17]. In light of these personal and interpersonal deficits in emotion recognition, it has been proposed that reduced ability to mentalize could be a key feature of ED psychopathology [34].

Yet, studies focused on mentalizing in BN are still scarce, and some research has failed to find mentalizing impairments in BN [50, 51]. In fact, some studies have shown bulimic patients to have average or even superior mentalizing abilities compared to healthy subjects [19, 43, 50, 55]. Accordingly, Pedersen et al. [50] reported a bimodal distribution in BN, suggesting two distinct subgroups within BN with regard to mentalizing (high vs. low abilities). Therefore, research on this topic might benefit from identifying additional factors that could account for this variability in mentalizing that has been observed in BN groups. In this respect, recent analyses have shown that up to 20% of ED patients also meet criteria for BPD [52]. Moreover, a larger proportion of patients, although not fulfilling BPD criteria, are deemed to present with BPD features [59]. However, no previous studies assessing mentalizing in BN have controlled for comorbid BPD symptoms. Given that BPD has been long associated with impaired mentalizing, it might be the case that this variable might contribute to some of the heterogeneity observed in previous results [3–5].

In this respect, recent lines of research challenged the use of the categorical diagnostic approach in relation to EDs. Instead, Q-sort analyses on large samples of patients discriminated separate trans-diagnostic ED profiles with distinct comorbidities and personality traits [60, 63]. According to this research, a consistent proportion of ED patients are characterized by high levels of impulsivity and BPD personality traits. However, other ED patients can show very different profiles with obsessive-compulsive and perfectionistic features or avoidant and depressive tendencies [60, 65].

Considering this heterogeneity of profiles and the above-mentioned relationship between BPD and impaired mentalizing, we hypothesize that BN subjects with lower levels of comorbid BPD symptoms will show better mentalizing, while those with higher levels of BPD comorbidity will have poorer mentalizing. This approach is consistent with both the categorical approach to EDs, separating patients into groups with different co-morbidities, and the phenomenological approach, in which a variety of symptoms, viewed as dimensions, reflect a common underlying pathology. In the present study, mentalizing, ED symptoms, BPD symptoms and general psychopathology were assessed in a sample of BN patients and healthy controls (HCs). We focused on several dimensions of mentalizing: uncertainty (hypomentalizing) and certainty (in the extreme hypermermentalizing) in interpreting mental states (Reflective Function Questionnaire); externally-based mentalizing such as others’ emotion recognition (Reading the Mind in the Eyes Test); and the level of differentiation and relatedness in descriptions of self and others (Differentiation-Relatedness Scale).

The following study hypotheses were tested:

1. BN patients would show lower scores in mentalizing measures compared to HCs. However, we expected differences between groups to decrease, when controlling for BPD features.
2. Lower scores in mentalizing in the BN sample were expected to be associated with higher ED symptoms severity. However, we also expected this relationship to level down when controlling for BPD features.
3. Finally, we tested a mediation model with BPD features as a mediator between Group (BN vs. HC) and mentalizing level.

## Methods

### Participants and procedures

The study protocol was reviewed and approved by the South East Research Ethics Committee (REC) and the London - Fulham NHS REC. In total 140 participants were included in the study with *n* = 53 BN patients and *n* = 87 HCs. Recruitment took place between July 2011 and May 2017. Part of the study population was assessed between 2011 and 2012 during a Randomized Clinical Trial (RCT) for evaluating the efficacy of a newly developed mentalization-based treatment (MBT) for EDs [53]. This subsample consisted of 43 BN patients and 62 HC. Data regarding BN patients were collected at baseline, before the beginning of the treatment. During the first half of 2017, an additional sample of 10 patients and 25 HC was recruited to increase the sample size based on a priori power analysis using G*Power 3.0.10 [30], which indicated that a total sample of *n* = 84 was needed to detect a large effect (Cohen’s d = .8) with 95% power, using a t test for independent samples with alpha at .05 (two tailed). The total number of participants in the present study was therefore *n* = 53 BN, *n* = 87 HC, *n* = 140 Total.

The clinical sample was recruited from three NHS trusts and consisted of outpatients between 18 and 55 years of age with a diagnosis of BN. Eating Disorder diagnoses were confirmed by means of the Eating Disorder Examination-Questionnaire (EDE-Q: [21]) according to DSM-5 diagnostic categories [1]. Borderline personality disorder was diagnosed using the SCID interview [32]. As questionnaires and interviews were conducted in English, fluency with the language was required as inclusion criteria. Additional exclusion criteria were a history or current diagnosis of Schizophrenia Spectrum And Other Psychotic Disorders, Autistic Spectrum Disorders (ASDs), severe brain damage and organic brain diseases leading to significant cognitive impairment. For the 43 subjects who were assessed during the first wave of testing (2011–2012), further inclusion criteria were the presence of suicidal or self-injurious behaviors and impulsivity in at least two areas (e.g. sexual behaviors, spending, substance abuse). As we intended to have a sample of BN with different degrees of BPD symptomatology, these criteria were not maintained for the second wave of testing (2017).

The HCs were recruited from University College London and from the community. Recruitment was carried out through the UCL Psychology Subject Pool as well as flyers and advertisements. Participants were asked to take part in the study if they had “no history of or current Eating Disorders or any mental health difficulty requiring treatment”. Moreover, participants with a BMI lower than 18.5 kg/m2 or not fluent in English were excluded from the sample. In addition, the last 25 HCs, corresponding to the second wave of testing, were administered the EDE-Q and the ZAN-BPD, and had their BMI measured to confirm the absence of ED and BPD diagnoses. Therefore, data regarding the BMI, EDE-Q and ZAN-BPD are available for only a portion of HCs representing 29% of the HC sample. However, measures were included in the analyses as post-hoc power calculations, based on the effect sizes found, revealed that results of the comparisons between groups were sufficiently powered (BMI: β < .02; EDE-Q: β < .001; ZAN-BPD: β < .001). All HCs were paid with a £10 Amazon voucher for their participation.

The BN sample was mostly (94.3%) female (F, *n* = 50; M, *n* = 3). The HCs contained 87.4% female participants (F, *n* = 76; M, *n* = 11). The distribution of gender between the two groups did not significantly differ, χ^2^ (1, *N* = 140) = 1.78, *p* = .24.

Before the beginning of the study, all participants received an information sheet explaining the goals and the procedure of the research. Eligible participants were then asked to provide written informed consent. Subsequently, a trained masters student administered to them the Demographics Questionnaire, EDE-Q, RFQ, RMET, Depression Anxiety Stress Scales (DASS-21), ZAN-BPD and ORI. The three descriptions of the ORI were recorded and transcribed. Afterwards, two independent assessors analysed the transcripts according to the DR-S. When scores were different, cases were discussed until agreement was reached. The inter-rater reliability between the two assessors was high with an average ICC of .93. Finally participants were debriefed and thanked.

However, not all the participants in the HC group completed the whole battery of tests assessing mentalizing. As testing was found distressing and a burden by some participants, during the first wave of testing the authors decided to drop some of the scales. The minimum number of HCs per each main outcome measures was 51. The number of participants who completed each measure is reported in the result section (Table 1).

**Table 1:** Means and standard deviations of the study variables, and between-group effect sizes

	Condition	N	Mean (SD)	95% Confidence Interval		
				Lower Bound	Upper Bound	Effect size
Age	BN	53	30.60 (8.91)	28.14	33.05	NS
	HC	87	29.14 (8.65)	27.30	30.98	
BMI	BN	53	23.55 (6.09)	21.87	25.23	NS
	HC	25	21.63 (2.72)	20.50	22.75	
EDE-Q	BN	53	4.47 (0.82)	4.24	4.70	4.01
	HC	25	0.94 (0.75)	0.63	1.25	
DASS-21	BN	53	38.26 (11.16)	35.18	41.34	3.89
	HC	50	9.62 (8.68)	7.15	12.09	
DR-S Self	BN	53	5.38 (1.04)	5.10	5.67	1.08
	HC	87	6.44 (0.92)	6.24	6.63	
DR-S Mother	BN	53	5.55 (1.08)	5.25	5.84	0.42
	HC	87	6.19 (0.82)	6.02	6.37	
DR-S Father	BN	53	5.90 (0.91)	5.65	6.15	0.45
	HC	87	6.31 (0.91)	6.12	6.50	
RMET	BN	53	26.09 (3.88)	25.02	27.16	0.62
	HC	51	28.27 (3.19)	27.38	29.17	
RFQ-C	BN	53	2.56 (2.53)	1.86	3.26	1.46
	HC	51	7.90 (4.76)	6.56	9.24	
RFQ-U	BN	53	8.90 (4.47)	7.67	10.13	1.90
	HC	51	1.90 (2.88)	1.09	2.71	
ZAN-BPD	BN	53	15.85 (5.45)	14.35	17.35	2.90
	HC	25	4.08 (2.67)	2.97	5.18	

Note. *BN* Bulimia Nervosa patients, *HC* Healthy controls. For EDE-Q, DASS-21, and ZAN-BPD total scores are reported

### Measures/instruments

#### Demographics questionnaire

Demographic information of participants was assessed by means of a preliminary questionnaire including gender, age, level of education, height, weight and psychiatric history.

#### Eating disorders examination-questionnaire (EDE-Q; [25, 26])

ED diagnosis and symptom severity were assessed using the EDE-Q which consists of a self-report 28 item questionnaire adapted from the Eating Disorders Examination interview (EDE; [29]). Participants were required to indicate the frequency to which they experienced ED symptoms over the previous four weeks. For the purposes of the current study, we focused on the EDE-Q Global score to assess the overall attitude towards food, body and weight of participants. The EDE-Q has been proven to have a good internal consistency (α = between .70 and 0.93; [9]). In this sample, for the Global EDE-Q score, we obtained a high level of internal consistency, α = .95.

#### Reflective function questionnaire (RFQ; [35])

The RFQ is a self-report questionnaire to assess capacity for mentalization in the context of attachment. It consists of 54 items that are rated on a 6-point Likert scale (from 1 = “strongly disagree” to 6 = “strongly agree”). Sample items include the following: “People’s thoughts are a mystery to me”, “I always know what I feel” and “I don’t always know why I do what I do”. Exploratory and confirmatory factor analyses in clinical and nonclinical samples yielded six items with high loading on two factors, namely “certainty about mental states” (RFQ-C) and “uncertainty about mental states” (RFQ-U; [34]). Thus, these two scales were used in the current study. Higher scores on the RFQ-C reflect the acknowledgement of mental states’ opaqueness and therefore a more genuine level of mentalizing. Conversely, lower scores are deemed to reflect hypermentalizing (the tendency to over-interpret mental states), which has been documented in patients with EDs [34]. On the other hand, the RFQ-U is used to assess of hypomentalizing (lack of knowledge about mental states). Specifically, higher scores on the RFQ-U are thought to reflect the tendency to think in concrete, non-mentalizing terms, while lower scores indicate better mentalizing [34]. In the current sample, the RFQ-U and the RFQ-C showed respectively a good (α = .76) and an excellent (α = .80) internal consistency.

#### Reading the mind in the eyes test-revised (RMET-R; [2])

This task includes 36 pictures of the eye region illustrating different emotionally charged or neutral mental states. Each picture is paired with four words representing different complex mental states (e.g., irritated, bored, ashamed). On a given trial, participants are asked to match each picture with the one word that better describes that mental state illustrated. The RMET-R is a performance-based measure of the level of Theory of Mind (ToM). Higher scores reflect higher ability to understand other people’s mental states, while lower scores indicate a deficit in this area. Previous research provided evidence for adequate test–retest reliability (ICC = .83) as well as internal reliability of this measure (α = .60; [38, 61]).

#### Differentiation-relatedness scale (DR-S; [24])

The DR-S is a procedure for assessing the level of integration, differentiation, and relatedness of self and significant others. The assessment is based on the *Object Relations Inventory* (ORI; [12]), a semi-structured interview for the assessment of object representations. The ORI consists in three open-ended descriptions of the mother, the father and the self. According to the DR-S, each description is then rated by an independent examiner on a 10-point scoring system, ranging from 1 to 10. Lower scores indicate primitive levels of object representations with compromised boundaries between the self and the others and an immature sense of interpersonal relatedness. Relationships are described primary in terms of the gratification or frustration, and there is little sense of the existence of others as separate entities independent to the subject [13]. Conversely upper scale levels reflect more complex and healthy object relations. Descriptions are more nuanced and coherent, reflecting an increasing capacity to integrate disparate aspects of self and other with increasing tolerance for ambivalence and ambiguity [13]. The instrument has been proven to be a valid and reliable measure of object representations [15, 18, 44]. The inter-rater reliabilities ranged from *r* = .80 to *r* = .99 [14, 16]. In the current sample, the internal reliability between the two assessors was proven to be high with an average ICC of .93.

#### Zanarini rating scale for borderline personality disorder (ZAN-BPD; [66])

The ZAN-BPD is a clinician-administered semi-structured interview for the assessment of BPD symptom severity. Each symptom domain is rated on a 5-point Likert Scale from 0 (no symptoms) to 4 (severe symptoms). The total score of ZAN-BPD provides a continuous measure of BPD psychopathology from a minimum of 0 to a maximum of 36. The scale has excellent internal reliability in non-clinical (α = 0.89) and clinical samples (α = 0.94; [34]).

#### Depression anxiety stress scales (DASS-21; [46])

The DASS-21 is a self-report questionnaire including 21 items tapping into three subscales: anxiety, depression and stress. For this study, we focused on the total score of the scale as a general measure of psychological distress. The DASS-21 has been shown to have high internal consistency (α = .93; [42]).

### Statistical analysis

Analysis of variance (ANOVA) was used to examine group differences with Group (BN vs. HC) as the independent variable and age, BMI, EDE-Q, DASS-21, ORI Self, ORI Mother, ORI Father, RMET, RFQ-C, RFQ-U and ZAN-BPD as dependent variables. Before analyzing between group differences, we performed Kolmogorov-Smirnov (K-S) and Levene’s tests to check for normality and variance homogeneity assumptions. Although some variables did not meet these assumptions, we used parametric tests as the overall F test is fairly robust against the violation of assumptions concerning normality [31]. Cohen’s *d* effect sizes were calculated as an additional index of between-group differences.

To take account of potential confounders for between groups differences in mentalizing, we intended to perform an analysis of covariance (ANCOVA) entering the total scores of the ZAN-BPD as a covariate. However, as the assumption of homogeneity of regression slopes was not met, ANCOVA could not be performed. ZAN-BPD scores showed a bimodal distribution with low scores characterizing HCs and high scores the BN sample. Indeed, the interaction term between ZAN-BPD and Group was found to be significant, *F*(1,101) = 2.38, *p* < .05. In order to further analyze the direction of this interaction, we performed two separate regressions for HCs and BN entering ZAN-BPD as the independent variable, and measures of mentalizing as dependent variables. Furthermore, we performed a t-test comparing mentalizing abilities in BN subjects with high and low BPD symptoms. To this aim, as suggested by previous literature, a score of 9 on the ZAN-BPD was used as cutoff [45]. Only 7 patients within the BN sample had a ZAN-BPD score < 9, while the vast majority (*n* = 53) had scores above the cut-off.

Pearson correlations were calculated to assess the relationship between the different variables within the BN sample. No subsequent regression analyses were performed as variables did not meet the assumptions of normality and multicollinearity.

Lastly, we tested mediation models entering Group as independent variable, measures of mentalizing as dependent variable, and ZAN-BPD scores as mediator. Mediation models were only tested for mentalizing measures that showed a significant correlation with ED (EDE-Q), BPD symptoms (ZAN-BPD) or with psychological distress (DASS-21) in the BN sample. As a result, three mediation models were tested, using the RFQ-C, RFQ-U and DR-S Self as dependent variables, respectively.

Statistical analyses were performed using SPSS software package Version 23. Mediation models were tested with the PROCESS macro for SPSS [41]. Missing values with a rate ≤ 20% per sample were estimated using the Expectation-Maximization method [62]. All statistical analyses were conducted two-tailed with an alpha level of .05.

## Results

Table 1 presents the means and standard deviations of all variables in the BN and the HC sample. The results of the ANOVA comparing the means in the two samples on the study variables are shown in Table 2. BN patients and HCs did not significantly differ in their age distribution and there was no statistical difference in BMI between both samples.

**Table 2:** One-way analysis of variance of demographic features, symptom severity and measures of mentalizing

	*df*	*SS*	*MS*	*F*	*d*	*p*
Age						
Between groups	1	70.22	70.22	.92	.17	.34
Within groups	138	10,553.47	76.47			
Total	138	10,623.70				
BMI						
Between groups	1	62.61	62.61	2.26	.41	.14
Within groups	76	2109.09	27.75			
Total	77	2171.70				
EDE-Q						<.001
Between groups	1	212.19	212.19	333.76	4.49	
Within groups	76	48.32	.64			
Total	77	260.51				
DASS-21			21,105.44	209.57		
Between groups	1	21,105.44	100.71		4.62	<.001
Within groups	76	10,171.28				
Total	77	31,276.73				
DR-S Self						
Between groups	1	36.52	36.52	39.00	1.08	<.001
Within groups	101	129.23	.94			
Total	102	165.75				
DR-S Mother						
Between groups	1	13.86	13.86	16.20	.67	<.001
Within groups	138	118.09	.86			
Total	139	131.95				
DR-S Father						
Between groups	1	5.52	5.52	6.66	.45	<.001
Within groups	138	114.24	.83			
Total	139	119.76				
RMET						
Between groups	1	123.92	123.92	9.78	.61	<.001
Within groups	101	1291.87	12.66			
Total	102	1415.79				
RFQ-C						
Between groups	1	741.69	741.69	51.52	1.40	<.001
Within groups	101	1468.32	14.39			
Total	102	2210.02				
RFQ-U						
Between groups	1	1272.71	1272.71	89.32	1.86	<.001
Within groups	101	1453.40	14.25			
Total	102	2726.11				
ZAN-BPD						
Between groups	1	2354.05	2354.05	104.20	2.74	<.001
Within groups	76	1717	22.59			
Total	77	4071.05				

Note. For EDE-Q, DASS-21, and ZAN-BPD total scores are reported

**Fig. 1:**
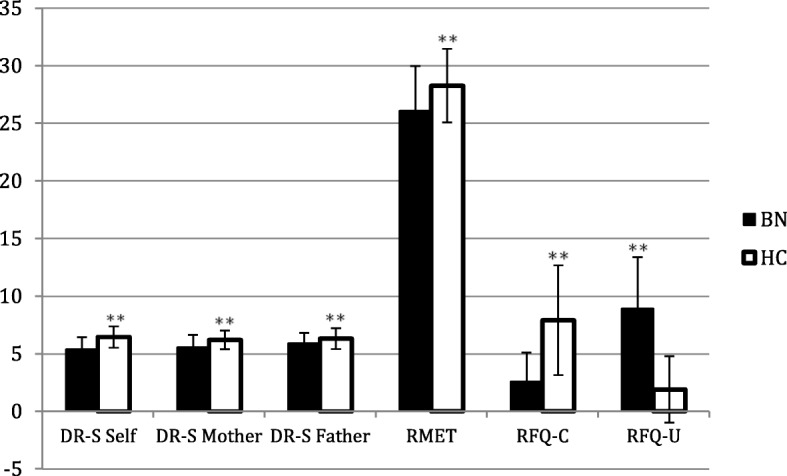
Means and standard deviations of the mentalizing measures in both BN and HCs (BN=Bulimia Nervosa patients, HC = Healthy controls). Mentalizing measures include the three subscales of the Differentiation-Relatedness Scale regarding the Self (DR-S Self), the Mother (DR-S Mother) and the Father (DR-S Father), the Reading The Mind in The Eyes Test (RMET), and the Reflective Function Questionnaires Certainty (RFQ-C) and Uncertainty (RFQ-U) subscales

Moreover, before testing the study hypotheses, we examined the extent to which BN patients and HCs differed in terms of ED symptoms (EDE-Q) and general psychological distress (DASS-21). As expected, BN patients were significantly higher in all subscales of the EDE-Q, with scores above the clinical threshold for each subscale. Conversely, controls showed a distribution of ED symptoms within the community norms [25]. As shown in Table 2, BN reported significantly elevated scores on the EDE-Q and DASS-21 compared to HCs. In addition, BN patients scored significantly higher on the total score of the ZAN-BPD. Moreover, as expected, BN patients had significantly lower scores on all measures of mentalizing compared to HCs (Fig. 1). Effect sizes were moderate to large.

Regarding the first hypothesis, we found that all measures of mentalizing in BN showed significantly lower scores compared to controls with moderate to large effect sizes (Table 1). In the second part of the first hypothesis we suggested that variation in the ZAN-BPD would account for at least some of the group effect. The Group x ZAN-BPD interaction was significant, and therefore it was not possible to perform an ANCOVA using ZAN-BPD as covariate. Subsequent linear regressions for HC and BN patients were performed to determine the nature of this interaction. In HCs, multivariate regression including all 6 mentalizing measures showed that the ZAN-BPD was not significantly associated with mentalizing, *F*(6,48) = 1.19, *p* = .26. However, when the RFQ-C and the RMET were tested separately, both were found to be significantly associated with the ZAN-BPD (*F*(8,16) = 5.13, *p* = < .01, and *F*(8,48) = 3.16, *p* = < .05). Specifically, higher scores on the ZAN-BPD were associated with lower scores on the RMET and on the RFQ-C. In BN patients, conversely the multivariate test was found to be significant (*F*(1,120) = 1.36, *p* < .05), indicating that higher scores on the ZAN-BPD were associated with worse mentalizing when all six measures of mentalizing were considered. Specifically, when looking at single effects, RFQ-U (*F*(20,32) = 2.01, *p* = < .05) and RMET (*F*(20,32) = 2.60, *p* = < .01) were significantly associated with the ZAN-BPD, with higher scores on the ZAN-BPD predicting lower scores on the RMET and higher scores on the RFQ-U.

In order to further analyse the influence of comorbid BPD symptoms on mentalizing, we compared BN patients with high and low ZAN-BPD scores (using a cut-off of 9; [45]) on all measures of mentalizing using t-tests. No significant differences between both groups of patients were found, with one exception. BN patients with low BPD symptoms scored significantly lower (*t*(51) = 2.61, *p* < .05) on the RFQ-U scale, indicating less impairments in mentalizing.

As for the second set of hypotheses, there were no significant correlations between ED symptom severity (EDE-Q Global score) and any of the measures of mentalizing. However, the EDE-Q Global score was significantly correlated with general psychological distress (DASS-21), *r* = .41, *p* < .01. Moreover, the DASS-21 was significantly correlated with the ZAN-BPD, *r* = .49, p < .01, and with two measures of mentalizing: the RFQ-U, *r* = .44, p < .01, and the RFQ-C, *r* = .30, *p* < .05. Also the ZAN-BPD was significantly correlated with two of the measures of mentalization: the RFQ-U, *r* = .37, *p* < .01, and the DR-S Self, *r* = −.32, *p* < .05.

**Fig. 2:**
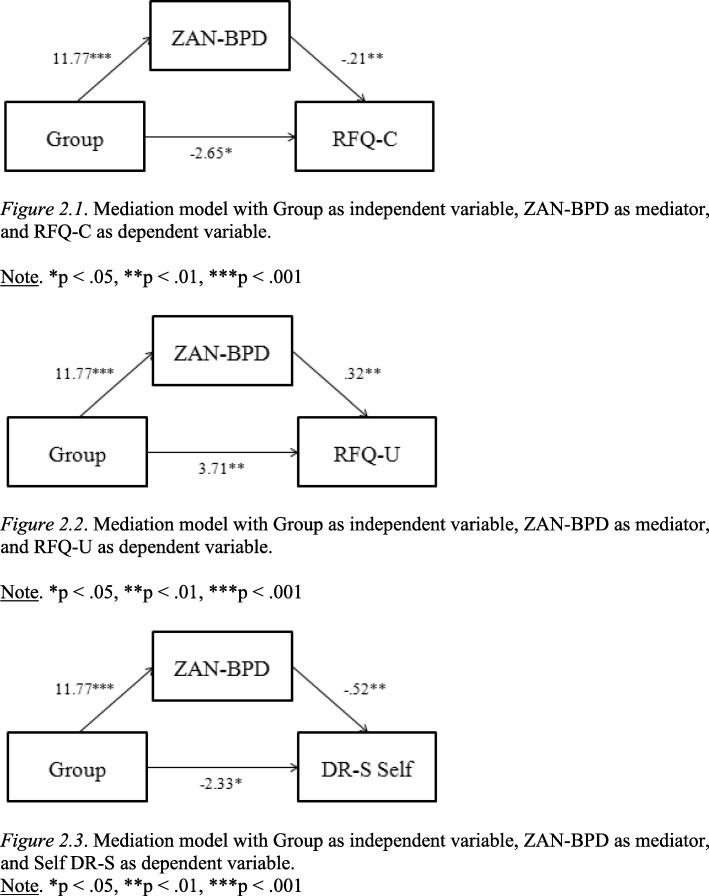
**1** Mediation model with Group as independent variable, ZAN-BPD as mediator, and RFQ-C as dependent variable. Note. *p < .05, **p < .01, ***p < .001. **2** Mediation model with Group as independent variable, ZAN-BPD as mediator, and RFQ-U as dependent variable. Note. *p < .05, **p < .01, ***p < .001. **3** Mediation model with Group as independent variable, ZAN-BPD as mediator, and Self DR-S as dependent variable. Note. *p < .05, **p < .01, ***p < .001

For testing the third study hypothesis, three mediation models were tested using Group as the independent variable, ZAN-BPD as the mediator, and RFQ-C, RFQ-U and DR-S Self respectively as dependent variables. As shown in Fig. 2 1, 2 and 3, both the direct and indirect effects of Group were significantly associated with poorer performances on mentalizing measures in all models.

## Discussion

The goal of the current study was to assess differences in mentalizing abilities between BN patients and HCs. The first hypothesis of the study was that BN subjects would have significantly lower levels of mentalizing. However, we expected that these differences in mentalizing would level down when adjusting for comorbid BPD symptoms. The first hypothesis was partially confirmed. BN patients showed significantly lower scores on all mentalizing measures (Fig. 1). However, it was not possible to control for the influence of BPD symptoms on group differences as the homogeneity of regression slopes assumption was not met.

In some previous studies, BN patients were found to exhibit deficits in social cognition and particularly in recognizing other people’s emotions through facial expressions as assessed by the RMET [17]. In line with those findings, the sample analyzed in the current study reported significantly lower scores on the RMET compared to HCs. Moreover, in the present investigation, deficits in symbolization and mentalizing were also demonstrated also in the ORI. When asked to describe themselves and their significant others, BN patients showed less complex object representations in relation to both the self and others. Descriptions were characterized by a stronger focus on physical appearance and body qualities rather than personality and psychological features. This is consistent with the observation that ED patients exhibit high levels of self-objectification and therefore have a tendency to evaluate oneself by physical versus non-physical means. In this respect, it could be speculated that self-objectification may be a subcomponent, or may arise as an epiphenomenon, of poor mentalizing. Boundaries between the self and others were found to be often compromised with a tendency to express polarized descriptions (characterized by idealization or denigration) without an attempt to integrate positive and negative aspects. These results are in accordance with a previous study that administered the ORI to a sample of mixed AN and BN patients finding impaired reflective symbolization of the self and others [56].

Lastly, BN patients scored significantly higher than HCs on the RFQ-U and significantly lower on the RFQ-C. Both of these differences suggest impaired mentalizing (hypomentalizing and hypermentalizing) in BN patients, indicating a lack of insight into the mental states of oneself and others.

These results seem to be in contrast with a previous study by Pedersen et al. [50] in which reflective functioning was analysed using the Reflective Functioning Scale (RF; [35]) in a sample of BN patients and HCs. In this study BN patients were found to have a more polarized pattern in their RF abilities with more scores both in the low and high range compared to HCs, who mainly reported scores in the medium range. These findings lead the authors to postulate that BN may develop and persist despite good mentalizing abilities. However, they also suggested that high RF scores could have resulted from raters mistakenly rating pseudomentalizing or hypermentalizing as high RF. This indeed remains a possibility as the RF scale does not adequately differentiate genuine RF from hypermentalizing. The RFQ used in the present study might be a better instrument for uncovering hypermentalizing and discrepancy of results between the two studies could therefore be better explained by the use of different instruments. The results presented in this report are, however, consistent with similar studies in patients with AN. Bers et al. [11] found significantly reduced agency, reflectivity and relatedness (but not differentiation) compared to HCs. Moreover, Bers et al. [10] using, as we did, the DR-S scale of the ORI in AN, found significantly reduced scores in descriptions of self, mother and father. This suggests that some aspects of mentalizing may be similarly impaired in both AN and BN, at least when the latter is complicated by BPD symptoms.

The second aim of the present study was to assess the degree to which mentalizing was associated with ED symptom severity in the BN sample. In this regard, previous studies have found a negative relationship between mentalizing and ED symptoms [56]. Yet, in this study, severity of ED symptoms was unrelated to mentalizing, suggesting that mentalizing abilities could be better conceptualized as a stable characteristic, independent of symptom severity.

Conversely, ED symptom severity was correlated with general psychological distress (DASS-21) which was correlated also with ZAN-BPD, suggesting that BN patients with more severe ED and BPD symptoms experience higher levels of psychological distress.

Furthermore, according to the third hypothesis of the study, BPD features were found to partially mediate the relationship between BN and indices of mentalizing (RFQ-U, RFQ-C, DR-S Self). However the direct effect of BN on mentalizing continued to be significant after controlling for ZAN-BPD score, indicating that both ED and BPD symptoms contribute independently in determining low mentalizing. The results support the hypothesis that impaired mentalizing is a characteristic of BN and not only a by-product of a comorbid symptomatology. However, current findings are limited by a relatively small sample of patients with a fairly homogenous high level of BPD features. Furthermore, for only three of the administered measures of mentalizing was it possible to test mediation models, restricting interpretation of the results to certain domains of mentalizing, namely reflective functioning and reflective symbolization of the self. The relationship between BN, BPD and other domains of mentalizing, such as social cognition and reflective symbolization of others, remained less clear. It is also a limitation that, for 62 of the 87 healthy controls, we relied on the participants denying any history of EDs, rather than using a structured ED instrument, as was done for the last 25 controls.

Alongside, future research should consider additional variables that can explain patients’ heterogeneity such as age of onset, illness duration, presence of diagnostic crossover, previous history of AN, and other comorbidities, especially mood disorders that have been previously associated with poor mentalizing [6, 33]. A possible association with mood received some support from our study, in which the DASS-21 scores showed a significant correlation with both RFQ scales. However, in the current study it was not possible to further investigate whether mood could account for part of the observed variability between groups in mentalizing. Indeed, DASS-21 did not meet assumptions for ANCOVA, and it was not possible to use this variable as covariate. Therefore, a possible direction for a further investigation would be to replicate the study design in a larger samples of BN and AN patients with and without BPD implementing the design with additional measures.

Moreover, the cross-sectional design of the study does not allow for inferences about causation but only associations. Whether low scores in mentalizing are a predisposing factor for BN or a by-product of the illness cannot be determined. Similarly, no conclusion can be drawn about the relationship between level of mentalizing and ED symptom severity over time. For example, it would be informative to analyze how both of these variables vary throughout treatment of BN. To address these issues, there is a need of larger scale studies with both community samples and currently ill patients, as well as studies employing longitudinal and prospective designs.

## Conclusion

Overall, the results of this study confirmed that patients with BN exhibit impaired mentalizing in regard to both the self and others. Furthermore, BPD features were found to play a role in explaining these impairments by partially mediating the relationship between BN and some indices of mentalizing. In conclusion, our findings lend empirical support for the hypothesis that impairments in mentalizing could be key in the etiology and maintenance of BN symptoms. It could be postulated that ED symptoms are used by patients as a maladaptive coping strategy to overcome emotional and interpersonal difficulties rising from the inability to understand behaviors in terms of mental states. Results of this study can thus be used to inform future directions of clinical practice. In this respect, BN patients could benefit from the development of treatment protocols aiming to implement mentalizing abilities. A randomized trial of Mentalization Based Therapy for Eating Disorders (MBT-ED) has been published with some promising results [53] and MBT-ED has been described in detail [54]. In turn, improving mentalizing could lead to better treatment outcomes and lower relapse rates. However, additional research will be needed to further validate this hypothesis.

## Abbreviations

APA: American Psychiatric Association
ASD: Autistic Spectrum Disorder
BMI: Body Mass Index
BN: Bulimia Nervosa
BPD: Borderline Personality Disorder
CBT-E: Enhanced cognitive behavioral therapy
DASS-21: Depression Anxiety Stress Scales
DBT: Dialectical behavioral therapy
DR-S: Differentiation-Relatedness Scale
DSM-5: Diagnostic and Statistical Manual of Mental Disorders-5
EDE-Q: Eating Disorder Examination-Questionnaire
MBT: Mentalization-based treatment
ORI: Object Relation Inventory
RCT: Randomized Clinical Trial
REC: Research Ethics Committee
RFQ: Reflective Function Questionnaire
RFQ-C: Reflective Function Questionnaire-Certainty
RFQ-U: Reflective Function Questionnaire-Uncertainty
RMET: Reading The Mind in The Eyes Test
ZAN-BPD: Zanarini Rating Scale for Borderline Personality Disorder
